# Nuclear Translocation of PFKFB3 Promotes Disuse‐Induced Muscle Atrophy via Scaffolding Nedd4‐Mediated JunB Ubiquitination

**DOI:** 10.1002/advs.77854

**Published:** 2026-09-16

**Authors:** Mengjun Ma, Yinliang Liu, Wangchang Wu, Biao Yang, Jiahao Zhuang, Haoye Yu, Rujia Mi, Yixuan Lu, Wen Yang, Hanting Yi, Chenglong Yuan, Yinfeng Gu, Hongyu Li

**Affiliations:** ^1^ Department of Orthopedics The Eighth Affiliated Hospital of Sun Yat‐Sen University Shenzhen China; ^2^ Biological Laboratory of Hetao Cooperation Zone the Eighth Affiliated Hospital of Sun Yat‐Sen University Shenzhen China; ^3^ Center For Biotherapy The Eighth Affiliated Hospital of Sun Yat‐Sen University Shenzhen China

**Keywords:** disuse, muscle atrophy, nuclear translocation, PFKFB3, ubiquitination

## Abstract

Disuse‐induced muscle atrophy is characterized by coordinated metabolic remodeling and enhanced protein degradation, yet the molecular link between these processes remains unclear. Here, we identify 6‐phosphofructo‐2‐kinase/fructose‐2,6‐bisphosphatase 3 (PFKFB3) as a critical regulator of this condition. Using a unilateral hindlimb immobilization mouse model, metabolomics, and functional assays in vivo and in vitro, we demonstrate that PFKFB3 is markedly upregulated during muscle atrophy and promotes myofiber wasting. Mechanistically, PFKFB3 predominantly localizes to the nucleus and functions independently of its canonical glycolytic activity. It acts as a scaffold protein to facilitate the interaction between the E3 ubiquitin ligase Nedd4 and the transcription factor JunB, thereby enhancing JunB ubiquitination and proteasomal degradation. Loss of JunB, a known anti‐atrophy factor, contributes to atrophic progression. In turn, JunB transcriptionally represses PFKFB3, forming a regulatory feedback loop. Pharmacological and genetic inhibition of the PFKFB3–Nedd4–JunB axis significantly attenuates muscle atrophy in vivo. Collectively, these findings reveal a noncanonical nuclear function of PFKFB3 in coordinating protein stability during muscle atrophy and highlight this signaling axis as a potential therapeutic target for disuse‐induced muscle wasting.

## Graphical abstract

Disuse‐induced muscle atrophy is driven by a non‐metabolic, nuclear function of the enzyme PFKFB3. Acting as a scaffold, PFKFB3 facilitates Nedd4‐mediated ubiquitination and degradation of the anti‐atrophy transcription factor JunB. Inhibiting this novel PFKFB3–Nedd4–JunB signaling axis stabilizes JunB and alleviates muscle wasting, revealing a highly promising therapeutic target for preventing atrophy.

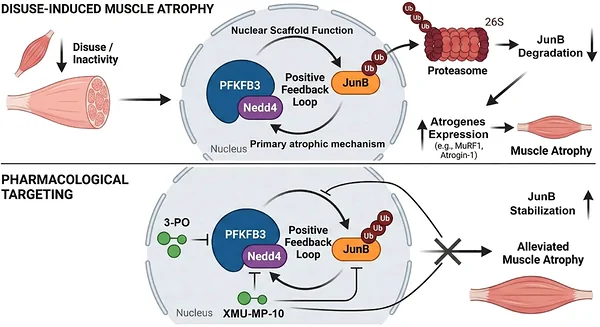

## 1 Introduction

Skeletal muscle atrophy is a multifactorial process characterized by progressive loss of muscle mass and function, frequently observed under conditions such as disuse, immobilization, and aging [1, 2, 3]. Although activation of proteolytic systems, particularly the ubiquitin–proteasome pathway, has been extensively implicated in muscle wasting, accumulating evidence suggests that metabolic reprogramming is also a critical determinant of atrophic progression [4, 5]. In particular, shifts from oxidative phosphorylation toward glycolytic metabolism have been reported in various models of muscle atrophy [6, 7, 8]; however, the molecular mediators linking metabolic remodeling to proteostasis remain incompletely understood.

6‐Phosphofructo‐2‐kinase/fructose‐2,6‐bisphosphatase 3 (PFKFB3) is a key regulator of glycolytic flux through its control of intracellular fructose‐2,6‐bisphosphate levels [9]. Beyond its canonical metabolic function, emerging studies have suggested that PFKFB3 may exert non‐metabolic roles, including regulation of cell cycle progression and transcriptional programs, particularly in the context of cancer and proliferative disorders [10, 11, 12]. Nevertheless, whether PFKFB3 contributes to skeletal muscle homeostasis, and more importantly, whether it participates in the pathogenesis of muscle atrophy, remains largely unexplored.

JunB, a member of the activator protein‐1 (AP‐1) transcription factor family, has been reported to promote muscle hypertrophy and antagonize atrophic signaling [13, 14]. Its protein stability is tightly controlled, yet the upstream mechanisms governing its degradation in skeletal muscle are not fully defined. Given that protein turnover is a central event in muscle atrophy [15, 16], elucidating how key transcriptional regulators such as JunB are modulated may provide mechanistic insight into disease progression.

In this study, we identify PFKFB3 as a previously underappreciated regulator of disuse‐induced muscle atrophy. We show that PFKFB3 is markedly upregulated in atrophic muscle and predominantly localizes to the nucleus [10, 17], where it exerts functions independent of its classical metabolic activity. Mechanistically, PFKFB3 directly interacts with JunB and promotes its ubiquitination and proteasomal degradation by facilitating the recruitment of the E3 ubiquitin ligase Nedd4. Furthermore, JunB reciprocally suppresses PFKFB3 transcription, forming a regulatory feedback loop. Functional analyses in vitro and in vivo demonstrate that disruption of this PFKFB3–Nedd4–JunB axis alleviates muscle atrophy. Collectively, these findings reveal a noncanonical role of PFKFB3 in coordinating metabolic signaling with protein homeostasis and highlight a potential therapeutic target for disuse‐induced muscle wasting.

## 2 Result

### 2.1 PFKFB3 Levels Are Associated with Disuse‐Induced Muscle Atrophy

**FIGURE 1:**
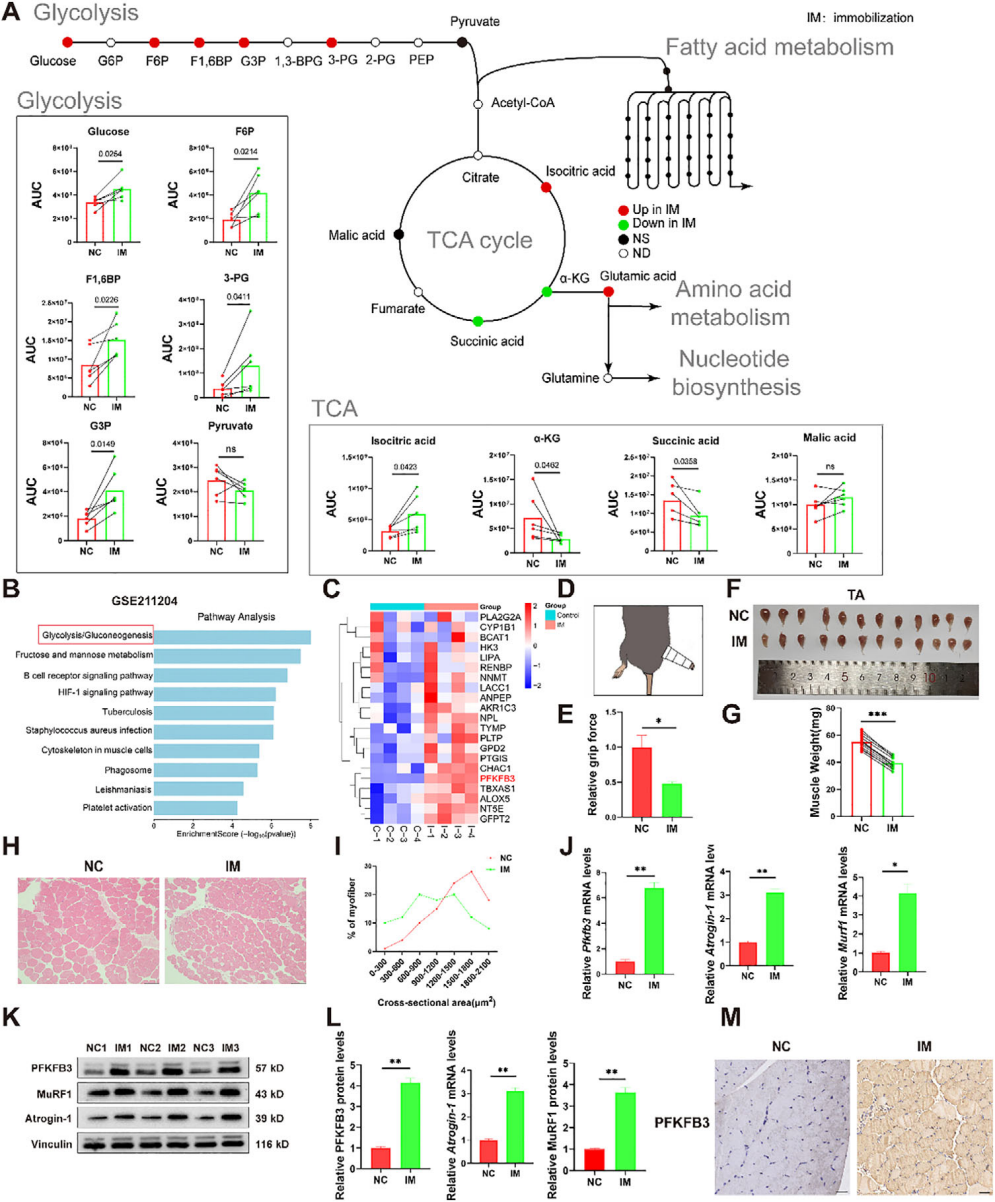
PFKFB3 is upregulated in disuse‐induced muscle atrophy. (A) Schematic summary of non‐targeted metabolomics analysis of tibialis anterior (TA) muscles from disuse‐induced muscle atrophy mice and control mice. Quantification bar plots are presented as mean ± SD, showing raw intensity values per biological replicate. Paired samples are connected by black lines. Differentially abundant metabolites are indicated as colored dots. n = 6. Adjusted p values were calculated using paired two‐tailed Student's t‐test or Wilcoxon test. NS, not significant; ND, not detected. (B) KEGG pathway enrichment analysis of differentially expressed genes from the GSE211204 dataset (log2FC (IM/Control) ≥ 1, p < 0.05). (C) Heatmap of differentially expressed genes involved in the glycolysis pathway. (D) Schematic diagram of the disuse‐induced muscle atrophy mouse model. The schematic diagram was generated by biorender. (E) Measurement of hindlimb grip strength in control and atrophy mice. (F) Representative images of TA muscles from control and atrophy mice (n = 12). (G) Quantification of TA muscle mass. (H–I) Hematoxylin and eosin (H&E) staining of TA muscle sections (H) and quantification of myofiber cross‐sectional area (CSA) (I). (J–L) mRNA (J) and protein levels (K–L) of MuRF1, Atrogin‐1, and PFKFB3 in mouse muscle tissues. Original blot can be found in Figure S12A–D. (M) Immunohistochemical staining of PFKFB3 in mouse muscle sections. All data are presented as mean ± SEM. *P* values: ^*^p < 0.05, ^**^p < 0.01, ^***^p < 0.001.

Metabolic alterations have always been a key factor in the development of muscle atrophy [18, 19]. To investigate metabolic changes in disuse‐induced muscle atrophy, we performed untargeted metabolomics analysis based on liquid chromatography–mass spectrometry (LC–MS) on tibialis anterior muscles from mice with disuse‐induced muscle atrophy and control muscles. The results suggested that glycolysis was significantly enhanced, whereas oxidative phosphorylation and the tricarboxylic acid (TCA) cycle were markedly reduced. The metabolism was reprogrammed from being mainly dependent on oxidative phosphorylation to being mainly dependent on glycolysis (Figure 1A).

By analyzing the public dataset GSE211204, we performed KEGG enrichment analysis on genes that were significantly upregulated after unilateral lower limb suspension in young healthy males. Among the enriched KEGG pathways, the most significant pathway was the glycolysis‐related pathway (Figure 1B). We then performed heatmap analysis on genes involved in glycolysis and found that the most prominently upregulated gene was PFKFB3 (6‐phosphofructo‐2‐kinase/fructose‐2,6‐bisphosphatase 3) (Figure 1C). Moreover, we found that the expression level of PFKFB3 was significantly decreased after recovery through voluntary exercise following suspension. These results suggest that PFKFB3 may be associated with disuse‐induced muscle atrophy (Figure S1A).

To verify this hypothesis, we established a mouse model of disuse‐induced muscle atrophy [2]. Twelve 6‐week‐old C57 mice were subjected to unilateral hindlimb immobilization (Figure 1D, Figure S1B). After one month, the grip strength of the hindlimbs was measured using a grip strength meter (Figure 1E), and then the mice were sacrificed. The tibialis anterior, gastrocnemius, and quadriceps muscles were collected. The left and right hindlimbs of the same mouse served as controls. Muscle size, weight, and cross‐sectional area were measured (Figure 1F–I, Figure S1C–J). Meanwhile, RNA and protein were extracted from the muscles, and the expression levels of the muscle atrophy‐related E3 ubiquitin ligases MuRF1 and Atrogin‐1 were detected by RT–qPCR and Western blot, respectively. The expression levels of MuRF1 and Atrogin‐1 were significantly upregulated in the experimental group, further confirming successful establishment of the disuse‐induced muscle atrophy model. At the same time, the expression level of PFKFB3 was examined and found to be significantly increased in atrophic muscles (Figure 1J–L). Immunohistochemical results also indicated that PFKFB3 expression was markedly increased under disuse‐induced muscle atrophy conditions (Figure 1M). These results indicate that PFKFB3 levels are significantly associated with disuse‐induced muscle atrophy.

In addition, by isolating muscle satellite cells and non‐satellite cells from mice, we found that there was no significant difference in PFKFB3 levels in satellite cells of atrophic mice, whereas a significant difference was observed in non‐satellite cells. This suggests that PFKFB3 may not regulate muscle atrophy by affecting satellite cell differentiation, but rather may directly affect muscle fibers (Figure S1K)

### 2.2 Muscle‐Specific Overexpression of PFKFB3 Induces and Aggravates Muscle Atrophy in Mice

**FIGURE 2:**
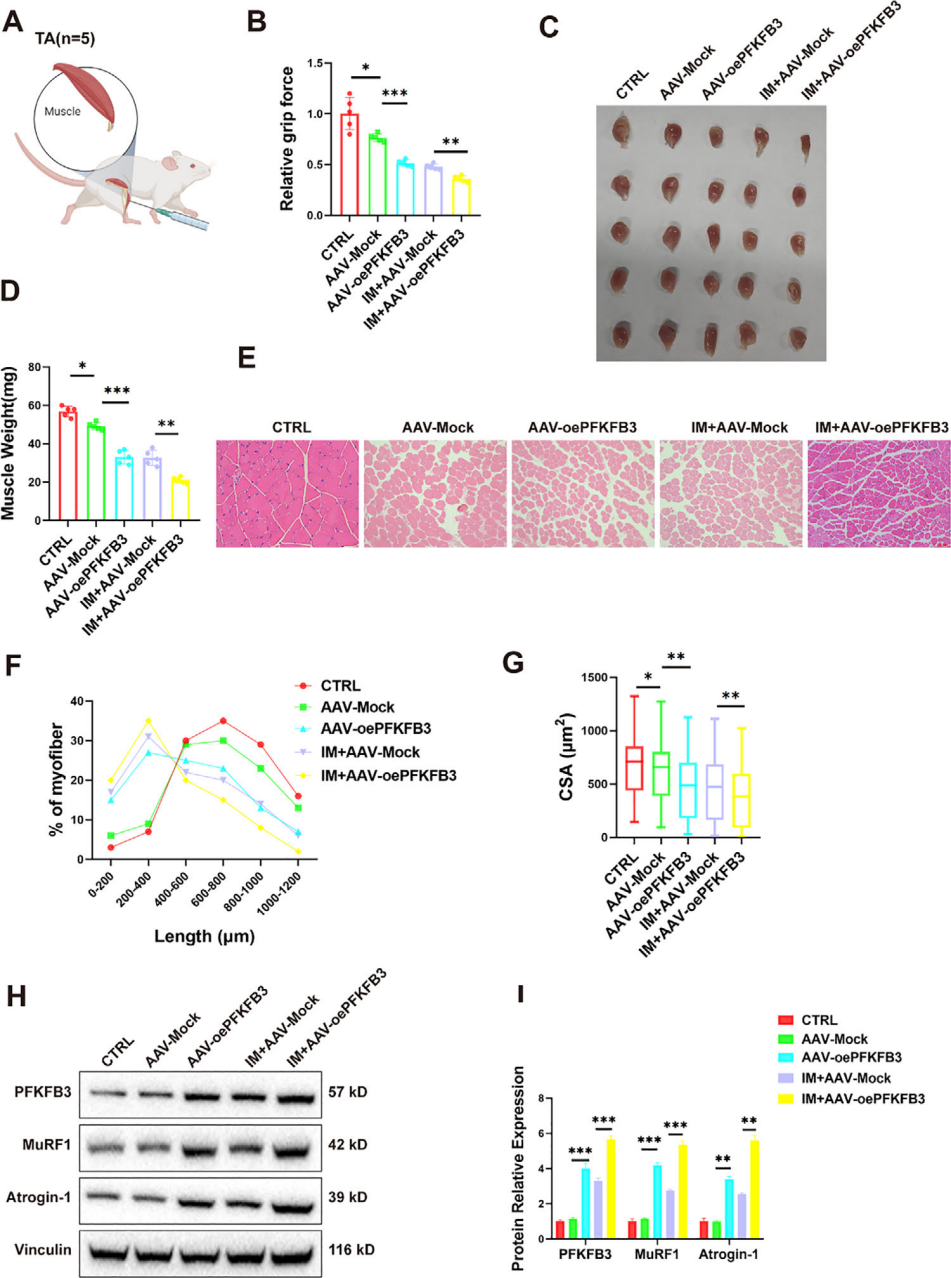
Muscle‐specific overexpression of PFKFB3 induces and exacerbates muscle atrophy. (A) Schematic diagram of AAV‐mediated overexpression of PFKFB3 in mouse tibialis anterior muscle (TA) (n = 5). The schematic diagram was generated by biorender. (B) The grip strength measurements of the mice in the control group (without AAV), AAV‐Mock, AAV‐oePFKFB3, IM + AAV‐Mock, and IM + AAV‐oePFKFB3 groups were conducted. (C) The tibialis anterior muscles of the control group (without AAV), AAV‐Mock, AAV‐oePFKFB3, IM + AAV‐Mock, and IM + AAV‐oePFKFB3 were examined. (D) The measurements of the mass of the anterior tibialis muscle in the control group (without AAV), the AAV‐Mock group, the AAV‐oePFKFB3 group, the IM + AAV‐Mock group, and the IM + AAV‐oePFKFB3 group. (E–G) H&E staining of TA muscle sections (E) and quantification of myofiber CSA (F‐G). (H, I) The protein expression levels of PFKFB3, MuRF1, and Atrogin‐1(H), along with their quantitative analyses (I), across the five experimental groups: Control (no AAV), AAV‐Mock, AAV‐oePFKFB3, IM + AAV‐Mock, and IM + AAV‐oePFKFB3. Original blot can be found in Figure S12E–H. All data are presented as mean ± SEM. *P* values: ^*^p < 0.05, ^**^p < 0.01, ^***^p < 0.001.

To investigate the role of PFKFB3 in mouse skeletal muscle, we constructed a muscle‐specific PFKFB3 overexpression model [20, 21]. We injected AAV9 adenovirus carrying PFKFB3 or control into the tibialis anterior muscle of 6‐week‐old C57BL/6 mice to generate tibialis anterior‐specific PFKFB3 overexpression mice (Figure 2A). One month after adenovirus injection, The grip strength measurements of the mice in the control group (without AAV), AAV‐Mock, AAV‐oePFKFB3, IM + AAV‐Mock, and IM + AAV‐oePFKFB3 groups were conducted. (Figure 2B). The tibialis anterior muscles were dissected from the Control (without AAV), AAV‐Mock, AAV‐oePFKFB3, IM + AAV‐Mock, and IM + AAV‐oePFKFB3 groups, and muscle mass was subsequently quantified. (Figure 2C–D). Then, we performed HE staining on the muscle sections of each group, and measured and statistically analyzed the cross‐sectional area of muscle fibers in each group. We found that overexpression of PFKFB3 significantly reduced the cross‐sectional area of muscle fibers (Figure 2E–G). We extracted proteins from the muscles of each group and measured the protein levels of PFKFB3, MuRF1, and Atrogin‐1. We found that PFKFB3 significantly increased the expression of the muscle atrophy‐related E3 ubiquitin ligases MuRF1 and Atrogin‐1(Figure 2H–I). These results indicate that muscle‐specific overexpression of PFKFB3 induces muscle atrophy in mice.

We further investigated the effects of PFKFB3 on fast muscle fibers and slow muscle fibers, and found that PFKFB3 had an impact on both types of fibers, causing their atrophy. Moreover, the atrophy was more pronounced in the fast muscle fibers (Figure S8A, B). These results indicate that PFKFB3 aggravates muscle atrophy.

To determine whether overexpression of PFKFB3 affects the fat infiltration of muscle fibers, we performed Oil Red O staining on frozen TA muscle sections. The results demonstrated no noticeable lipid accumulation in the interstitial areas around the myofibers, confirming that PFKFB3 overexpression does not induce intramuscular ectopic fat deposition (Figure S11A).

### 2.3 PFKFB3 Induces C2C12 Myotube Atrophy in Vitro

**FIGURE 3:**
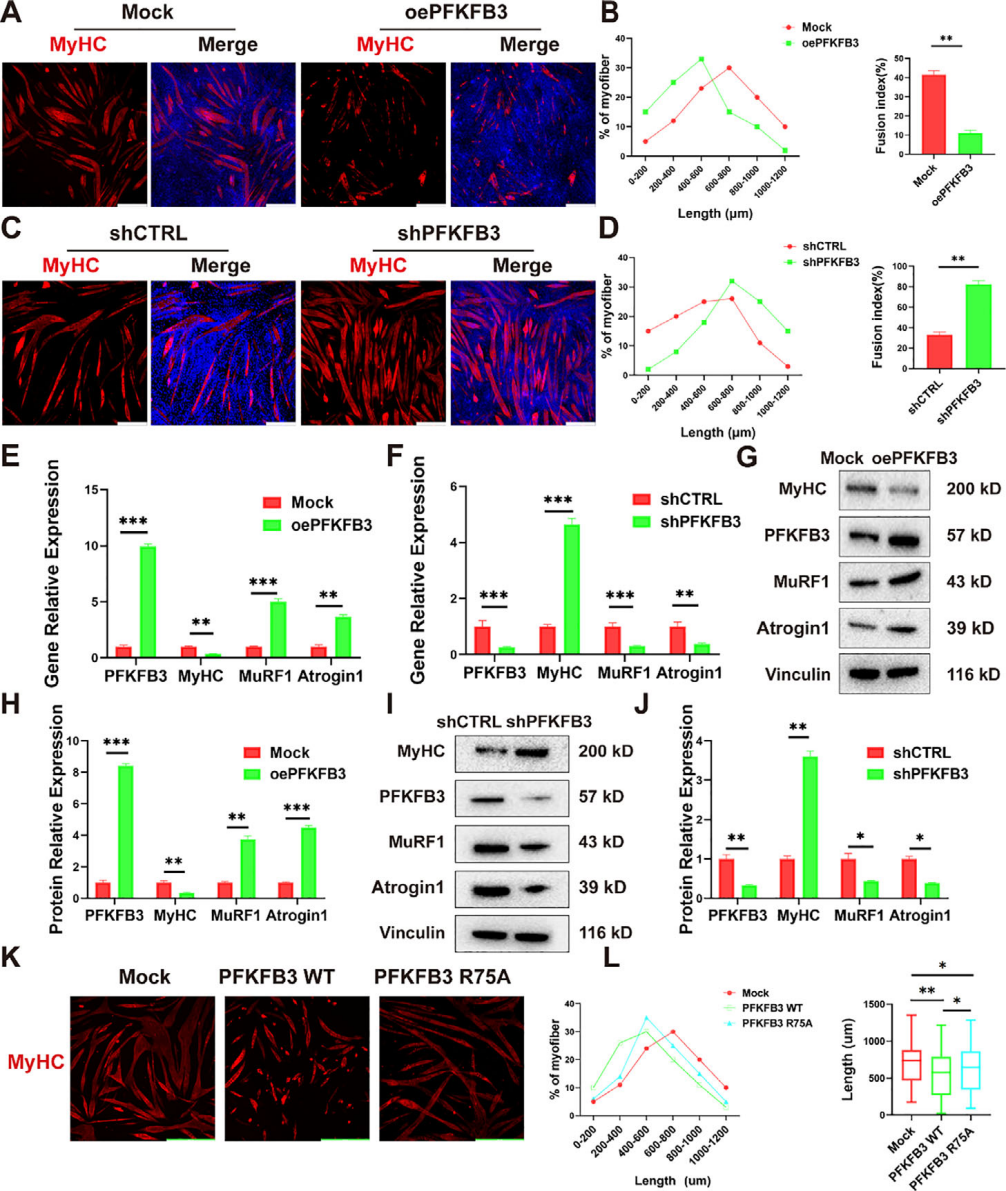
PFKFB3 induces atrophy in C2C12 myotubes. (A) Immunofluorescence staining of C2C12 myotubes overexpressing PFKFB3. (B) Quantification of myotube length and fusion index compared to control. (C) Immunofluorescence staining of C2C12 myotubes with PFKFB3 knockdown. (D) Quantification of myotube length and fusion index. (E, F) mRNA levels of MyHC, MuRF1, and Atrogin‐1 in PFKFB3‐overexpressing (E) and knockdown (F) myotubes. (G–J) Protein levels of MyHC, MuRF1, and Atrogin‐1 in PFKFB3‐overexpressing (G, H) and knockdown (I, J) groups. Original blot can be found in Figure S12I–M and Figure S13A–E. (K, L) Immunofluorescence staining of MyHC (K) and quantification of myotube length (L) in 3‐PO‐treated vs. PFKFB3‐overexpressing myotubes. All data are presented as mean ± SEM. *P* values: ^*^p < 0.05, ^**^p < 0.01, ^***^p < 0.001.

To investigate the effect of PFKFB3 on C2C12 myotubes, we constructed C2C12 cell models with oePFKFB3 and shPFKFB3. We found that C2C12 myotubes in the oePFKFB3 group were significantly shorter and thinner compared with the control group, and the fusion index was also significantly lower (Figure 3A, B). In contrast, C2C12 myotubes in the shPFKFB3 group were significantly longer and thicker, and the fusion index was significantly higher (Figure 3C, D). RT–qPCR and Western blot were used to verify the expression of PFKFB3, as well as MyHC and the atrophy‐related E3 ubiquitin ligases MuRF1 and Atrogin‐1. We found that overexpression of PFKFB3 reduced MyHC expression and significantly increased MuRF1 and Atrogin‐1 expression (Figure 3E–G), whereas knockdown of PFKFB3 showed the opposite effect (Figure 3H–J). These results indicate that PFKFB3 induces C2C12 myotube atrophy in vitro. Considering that LV and AdV can typically independently induce the ubiquitin‐proteasome system (UPS) regardless of any other sequences, and significantly increase the expression levels of MuRF1 and MAFbx. We conducted additional experiments with AAV in C2C12 cells and set up corresponding virus‐free control groups to further clarify our conclusions (Figure S10A).

Whether PFKFB3‐induced myotube atrophy is due to its classical metabolic enzyme function remains unclear. To verify this hypothesis, we simultaneously constructed a kinase inactivation mutant PFKFB3 R75A and treated the C2C12 myotube cells transfected with oePFKFB3 with the small molecule inhibitor 3‐PO (which can inhibit the enzymatic activity of PFKFB3) [22]. Compared with the PFKFB3 WT group, the PFKFB3 R75A group partially reversed the muscle atrophy, indicating that the kinase activity played a certain role in the muscle atrophy caused by PFKFB3. However, the PFKFB3 R75A group was unable to completely reverse the muscle atrophy, indicating that there are other mechanisms besides enzyme activity that contribute to the occurrence of muscle tube atrophy (Figure 3K–L). The results of the 3‐PO treatment group and other experiments also support this conclusion (Figure S2A, B). QPCR and Western blot results also indicated partial improvement but not complete reversal (Figure S2C–E). These results suggest that the mechanism by which PFKFB3 induces C2C12 myotube atrophy is not solely dependent on its classical metabolic enzyme function, but may involve other mechanisms [9].

### 2.4 PFKFB3 Mainly Functions in the Nucleus to Induce Muscle Atrophy

**FIGURE 4:**
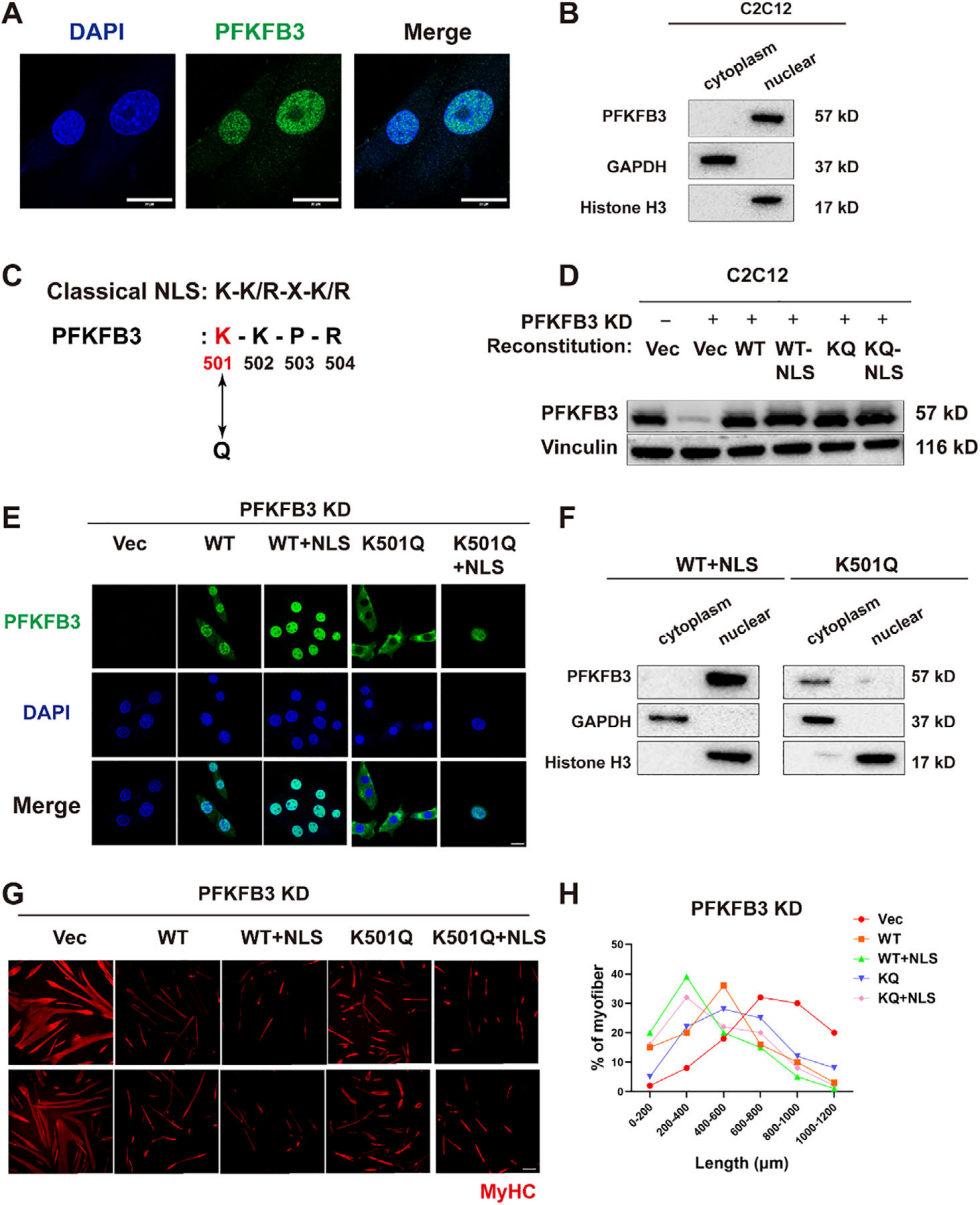
Nuclear PFKFB3 predominantly mediates myotube atrophy. (A) Immunofluorescence localization of PFKFB3 in C2C12 cells. (B) Nuclear and cytoplasmic fractionation of C2C12 cells. Original blot can be found in Figure S13F–H. (C) Schematic of the canonical nuclear localization sequence (NLS) and NLS‐mutant constructs of PFKFB3. (D) Expression levels of PFKFB3 in different C2C12 models. Original blot can be found in Figure S13I, J. (E) The results of immunofluorescence staining for PFKFB3 in C2C12 cells under each experimental condition. (F) Nuclear–cytoplasmic fractionation and Western blot analysis in WT+NLS and K501Q groups. Original blot can be found in Figure S13K–P. (G, H) MyHC immunofluorescence staining (G) and quantification of myotube length (H).

It is noteworthy that immunofluorescence analysis of human muscle tissue sections showed that PFKFB3 is mainly localized in the nucleus of muscle fibers (Figure S3A). We also found that PFKFB3 is predominantly localized in the nucleus of C2C12 cells (Figure 4A), which is different from the conventional understanding that PFKFB3, as a glycolytic enzyme, is mainly localized in the cytoplasm. Nuclear–cytoplasmic fractionation followed by Western blot further confirmed that PFKFB3 is mainly localized in the nucleus (Figure 4B). We speculated that PFKFB3 may function in the nucleus of C2C12 cells. Unlike other PFKFB enzymes that are localized in the cytoplasm, PFKFB3 is mainly localized in the nucleus. PFKFB3 contains a classical nuclear localization signal (NLS) as “KKPR” (amino acids 501–504) [17]. To verify the functional localization of PFKFB3, we constructed an NLS‐mutant C2C12 cell line (K501Q) (Figure 4C). In order to further investigate the importance of nuclear localization of PFKFB3, an additional NLS (sequence as “PKKKRKVD”) that is different from PFKFB3‐NLS was added to both the N and C terminus of WT or mutant PFKFB3 (namely WT+NLS and K501Q+NLS, respectively). The two mutants were then stably expressed in PFKFB3 knockdown cells, and IF results confirmed that additional NLS retained both WT and K501Q PFKFB3 in the nucleus (Figure 4D, E).

Nuclear–cytoplasmic fractionation and Western blot were performed to detect nuclear and cytoplasmic PFKFB3 expression (Figure 4F). Immunofluorescence was used to assess myotube length and thickness (Figure 4G, H). In addition, qPCR and Western blot were used to detect MyHC, MuRF1, and Atrogin‐1 expression (Figure S3B, C). The results showed that when PFKFB3 was restricted to the nucleus, C2C12 myotube atrophy was most severe, whereas when PFKFB3 was localized only in the cytoplasm, the atrophy phenotype was less pronounced. These results indicate that PFKFB3 mainly functions in the nucleus to induce muscle atrophy.

### 2.5 PFKFB3 Regulates Muscle Atrophy by Modulating JunB Expression

**FIGURE 5:**
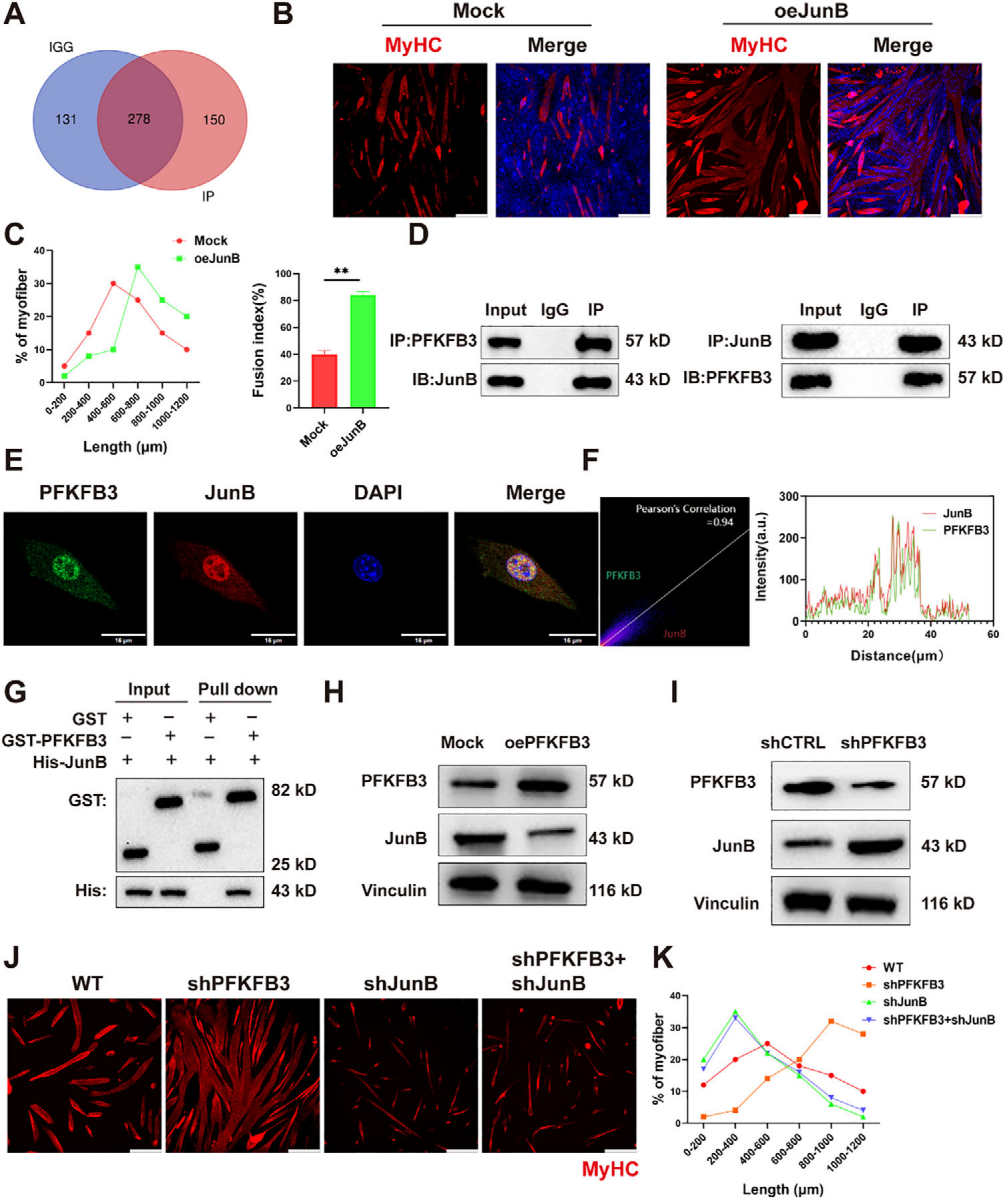
PFKFB3 regulates muscle atrophy via the transcription factor JunB. (A) Venn diagram showing overlap between proteins identified by IP‐MS in IP and IgG groups. (B, C) MyHC immunofluorescence staining (B) and quantification of myotube length and fusion index (C) upon JunB overexpression. (D) Co‐immunoprecipitation (Co‐IP) showing interaction between PFKFB3 and JunB. Original blot can be found in Figure S14A–D. (E, F) Confocal immunofluorescence colocalization (E) and correlation analysis (F). (G) GST pull‐down assay demonstrating direct interaction. Original blot can be found in Figure S14E, F. (H, I) Effects of PFKFB3 overexpression or knockdown on JunB protein levels. Original blot can be found in Figure S14G–L. (J, K) MyHC staining (J) and myotube length quantification (K).

To further explore the molecular mechanism by which PFKFB3 induces muscle atrophy, we performed immunoprecipitation (IP) experiments using an antibody against PFKFB3 to pull down interacting proteins. Coomassie brilliant blue staining was first used to preliminarily assess whether interacting proteins were present (Figure S4A). The samples were then subjected to IP–MS analysis to identify potential interacting proteins. The mass spectrometry results showed that 150 proteins were specifically enriched in the IP group (Figure 5A). Among these proteins, we screened for those mainly localized in the nucleus and directly related to skeletal muscle atrophy, and identified JunB. JunB is a transcription factor belonging to the AP‐1 family, and previous studies have shown that JunB promotes skeletal muscle hypertrophy and is closely associated with muscle atrophy [13]. We further validated this by constructing oeJunB and shJunB cell models, confirming that JunB promotes hypertrophy and inhibits muscle atrophy (Figure 5B, C, Figure S4B–I).

Whether PFKFB3 regulates muscle atrophy through JunB remained to be determined. Mass spectrometry results suggested that JunB may interact with PFKFB3. Co‐immunoprecipitation (Co‐IP) experiments further confirmed that PFKFB3 binds to JunB (Figure 5D), and immunofluorescence analysis demonstrated clear co‐localization of PFKFB3 and JunB in C2C12 cells (Figure 5E, F). In addition, in vitro GST pull‐down assays confirmed the direct interaction between PFKFB3 and JunB (Figure 5G).

To further investigate the regulatory relationship between PFKFB3 and JunB, Western blot analysis showed that overexpression of PFKFB3 significantly reduced JunB protein levels, whereas knockdown of PFKFB3 increased JunB expression (Figure 5H–I, Figure S5A, B). These results suggest that PFKFB3 may participate in the process of muscle atrophy by directly binding to and regulating the expression of JunB.

To verify whether PFKFB3 regulates muscle atrophy through JunB, we constructed shPFKFB3 + shJunB C2C12 cell models. The results showed that knockdown of JunB significantly suppressed the myotube hypertrophy induced by PFKFB3 knockdown (Figure 5J–K). In addition, knockdown of JunB significantly reduced MyHC levels (Figure S5C, D). These results indicate that PFKFB3 regulates myotube atrophy by decreasing JunB protein levels. We have validated the temporal dynamics of the reciprocal regulation between PFKFB3 and JunB (Figure S5E).

### 2.6 PFKFB3 Promotes Ubiquitination and Degradation of JunB

**FIGURE 6:**
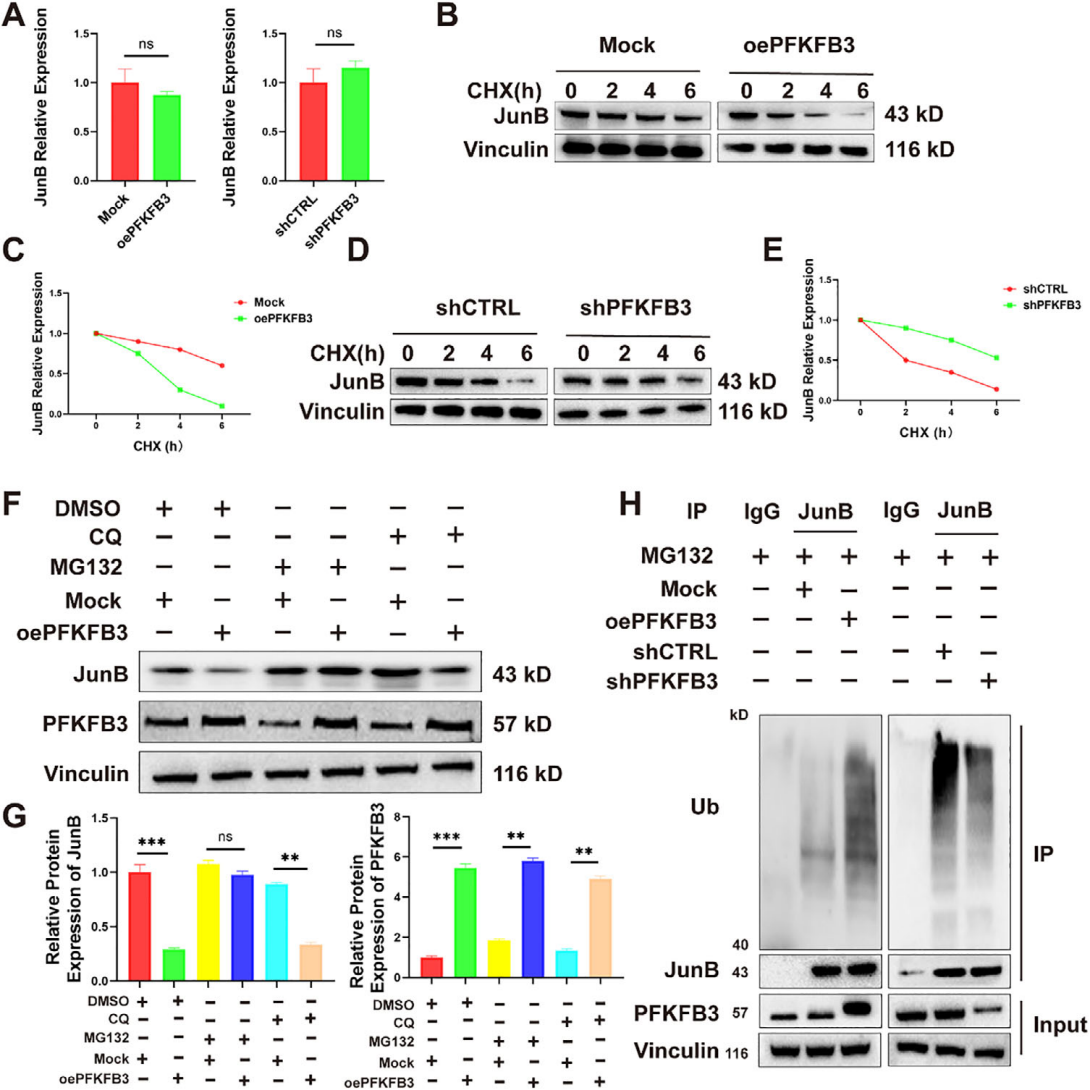
PFKFB3 promotes ubiquitin‐mediated degradation of JunB. (A) The effects of overexpression of PFKFB3 and knockdown of PFKFB3 on the RNA level of JunB. (B, C) Cycloheximide (CHX) chase assay and quantification in PFKFB3‐overexpressing cells. Original blot can be found in Figure S14M–P. (D, E) CHX assay and quantification in PFKFB3 knockdown cells. Original blot can be found in Figure S15A‐D. (F, G) Western blot and quantification of JunB protein levels after treatment with MG132 or chloroquine (CQ). Original blot can be found in Figure S15E–G. (H) Using the antibody of JunB for IP experiments, combined with the JunB protein, the ubiquitin levels of JunB were respectively detected when PFKFB3 was overexpressed or knocked down. Original blot can be found in Figure S15H–O. All data are presented as mean ± SEM. NS, not significant; ^*^p < 0.05, ^**^p < 0.01, ^***^p < 0.001.

We further explored the specific molecular mechanism by which PFKFB3 regulates JunB. Previous results showed that PFKFB3 affects JunB protein levels; therefore, we next examined changes at the mRNA level using RT–qPCR. Notably, PFKFB3 did not affect JunB mRNA levels (Figure 6A), suggesting that the regulation occurs at the protein level. Cycloheximide (CHX) chase experiments showed that PFKFB3 significantly shortened the half‐life of JunB protein and accelerated its degradation (Figure 6B–E). To further determine the degradation pathway, we treated cells with MG132 (a ubiquitin–proteasome pathway inhibitor) and CQ (an autophagy–lysosome pathway inhibitor). The results showed that MG132 treatment inhibited PFKFB3‐mediated degradation of JunB, whereas CQ treatment did not block this effect (Figure 6F–G). These results indicate that PFKFB3 promotes JunB degradation through the ubiquitin–proteasome pathway rather than the autophagy–lysosome pathway.

Furthermore, IP and Western blot assays were used to assess the ubiquitination level of JunB. Overexpression of PFKFB3 significantly increased JunB ubiquitination, whereas knockdown of PFKFB3 decreased JunB ubiquitination (Figure 6H). These findings demonstrate that PFKFB3 promotes ubiquitin‐mediated degradation of JunB.

### 2.7 Identification of Nedd4 as an E3 Ubiquitin Ligase for JunB

Given that PFKFB3 promoted the ubiquitination and degradation of JunB, we wondered whether PFKFB3 was a novel E3 ubiquitin ligase for JunB. However, we found no canonical catalytic structure of E3 ubiquitin ligase in the domain of PFKFB3 by analyzing the amino acid sequence. Hence, we hypothesized that PFKFB3 regulated the ubiquitination level of JunB by relying on an E3 ubiquitin ligase. To verify this hypothesis, we retrieved the UbiBrowser database (http://ubibrowser.ncpsb.org/) and screened the potential E3 ubiquitin ligases of JunB [23].

**FIGURE 7:**
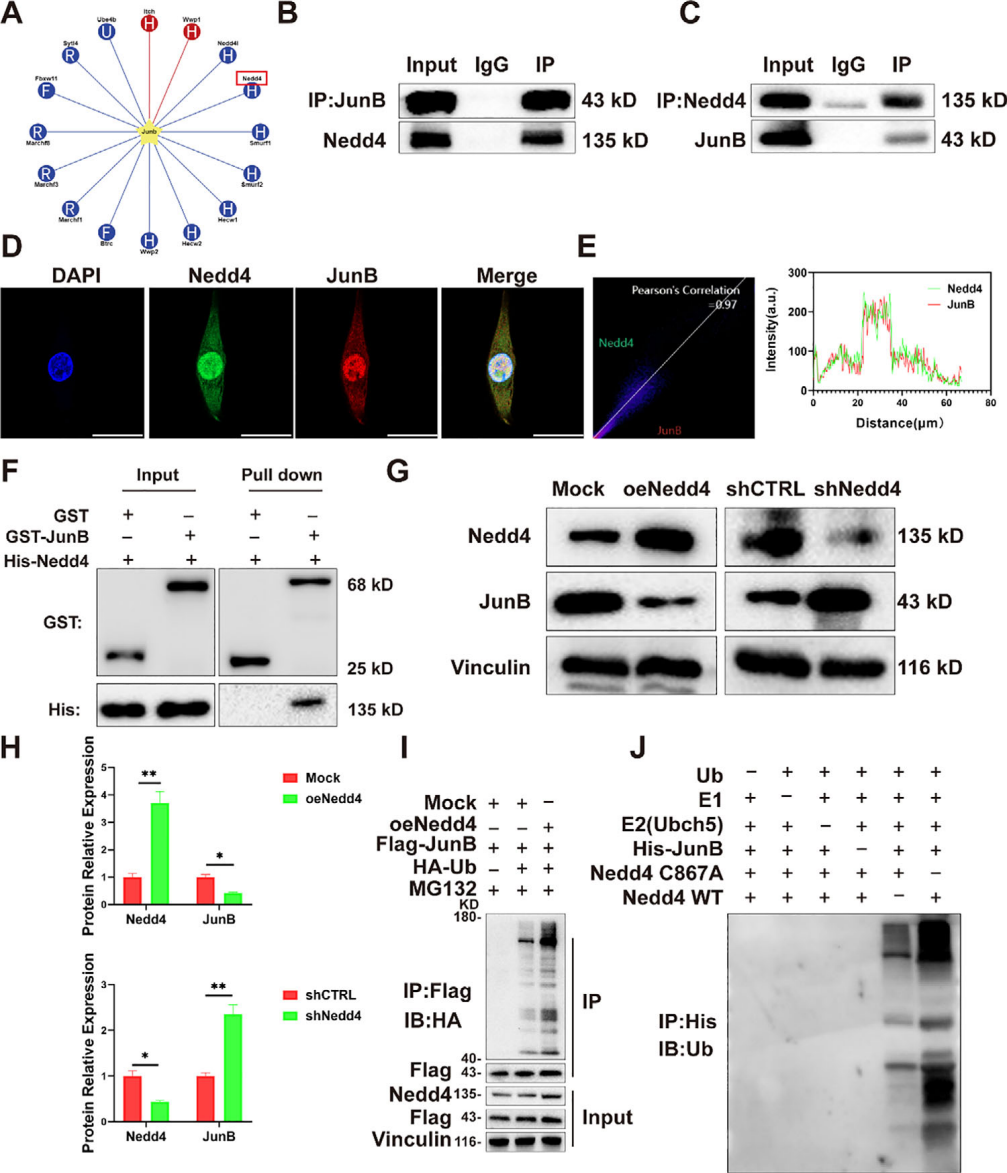
Nedd4 is identified as an E3 ubiquitin ligase for JunB. (A) Prediction of JunB E3 ligases using the UbiBrowser database. (B, C) In C2C12 cells, the antibody of JunB was used as the IP antibody to detect the protein level of Nedd4. The Nedd4 antibody was used as an IP antibody to detect the protein level of JunB. Original blot can be found in Figure S16A–D. (D–E) Immunofluorescence staining was performed simultaneously on Nedd4 and JunB in C2C12 cells, and co‐localization and related analysis were conducted. (F) GST pull‐down assay. The GST‐tagged JunB protein was expressed in Escherichia coli and purified using glutathione‐S‐ubiquitin resin. The cell lysates containing potential interacting proteins were incubated with the immobilized GST fusion protein. Original blot can be found in Figure S16E, F. (G, H) JunB protein levels and quantification upon Nedd4 overexpression or knockdown. Original blot can be found in Figure S16G‐I. (I) Flag‐JunB and HA‐Ub were respectively co‐transfected into 293T cells. An IP experiment was conducted using Flag antibodies to detect the levels of HA in the overexpressed Nedd4 group and the control group. Original blot can be found in Figure S16J–N. (J) In vitro ubiquitination reconstitution experiment, which involved using the purified components of the recombinant protein (E1, UbcH5 family E2, wild‐type Nedd4 and its C867A catalytically inactive mutant, His‐tagged JunB, and ubiquitin). Original blot can be found in Figure S17A. All data are presented as mean ± SEM. NS, not significant; ^*^ *p* < 0.05, ^**^ *p* < 0.01, ^***^ *p* < 0.001.

We then found that among the proteins specifically interacting with PFKFB3 in the IP–MS results, there was an E3 ubiquitin ligase, Nedd4. Moreover, Nedd4 was also predicted as a highly probable E3 ubiquitin ligase for JunB in the UbiBrowser database (Figure 7A). Hence Nedd4 was regarded as the target of our research. Moreover, we showed that Nedd4 interacted with JunB both at the exogenous and endogenous level.

Immunofluorescence analysis further confirmed that Nedd4 and JunB exhibited clear co‐localization in C2C12 cells (Figure 7B–F). Therefore, these results demonstrated that Nedd4 was a candidate E3 ubiquitin ligase for JunB. Notably, we found that Nedd4 regulated the protein level but not the mRNA level of JunB (Figure 7G, H, Figure S6A, B). Nedd4 significantly increased the ubiquitination level of JunB, and knockdown of Nedd4 significantly decreased JunB ubiquitination (Figure 7I, Figure S6C). We conducted an in vitro ubiquitination reconstitution experiment, which involved using the purified components of the recombinant protein (E1, UbcH5 family E2, wild‐type Nedd4 and its C867A catalytically inactive mutant, His‐tagged JunB, and ubiquitin) to verify that Nedd4 directly catalyzes the ubiquitination and degradation of JunB (Figure 7J). Collectively, these findings strongly suggested that Nedd4 was an E3 ubiquitin ligase for JunB.

### 2.8 PFKFB3 Acts as a Scaffold Protein Bridging JunB and Nedd4

**FIGURE 8:**
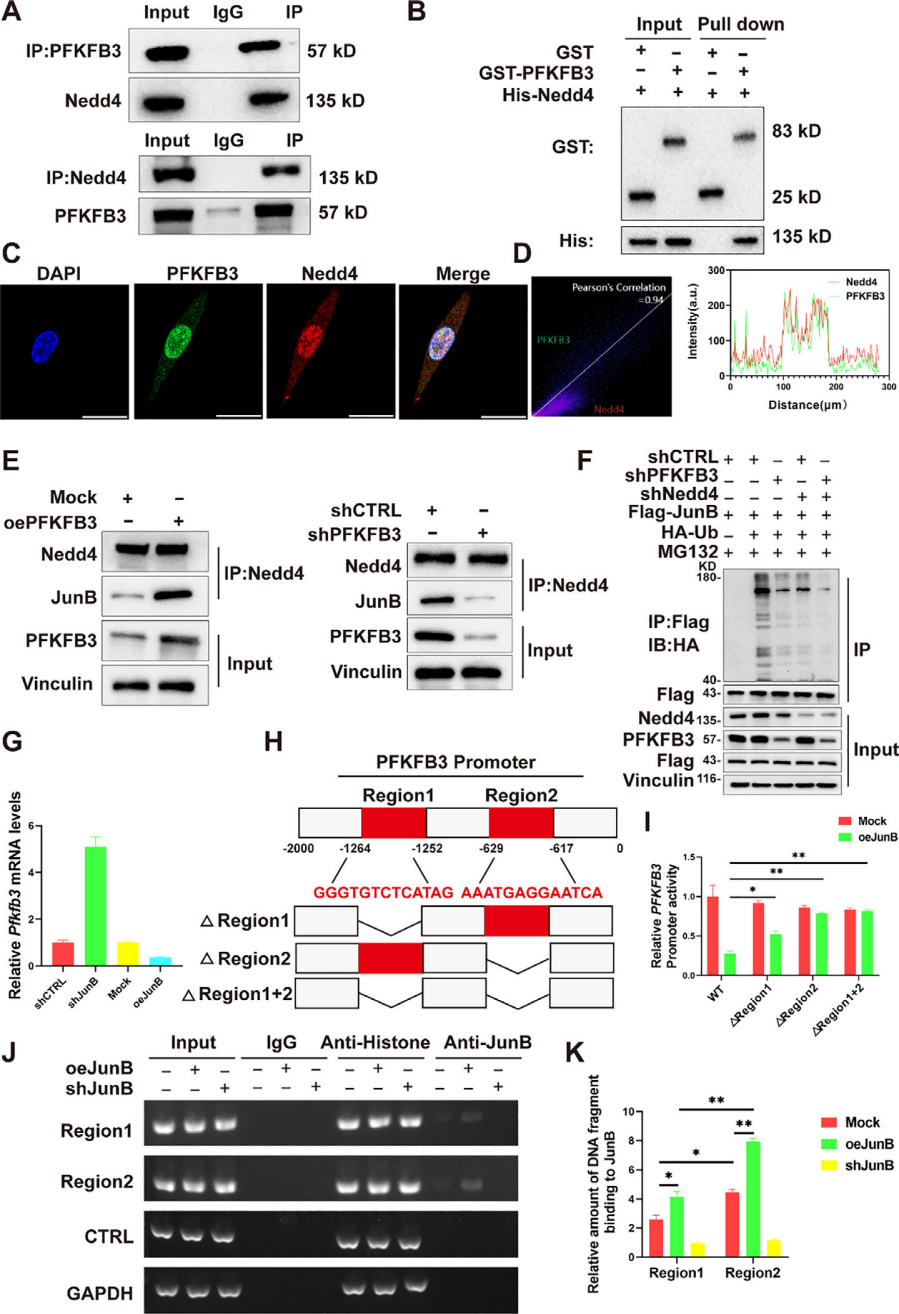
PFKFB3 acts as a scaffold to facilitate Nedd4–JunB interaction. (A) Co‐IP assays showing interactions among PFKFB3, Nedd4, and JunB. Original blot can be found in Figure S17B–E. (B) GST pull‐down assay between PFKFB3 and Nedd4. Original blot can be found in Figure S17F, G. (C, D) Confocal colocalization and correlation analysis. (E) Effect of PFKFB3 expression on Nedd4–JunB interaction. Original blot can be found in Figure S17H–O. (F) Effects of PFKFB3 or Nedd4 knockdown on JunB ubiquitination. Original blot can be found in Figure S17P‐U. (G) Effects of JunB on PFKFB3 mRNA levels. (H) Schematic of predicted JunB‐binding sites in the PFKFB3 promoter and mutant constructs. (I) Luciferase reporter assay for PFKFB3 promoter activation by JunB. (J, K) ChIP‐PCR (J) and qPCR quantification (K) validating JunB binding. All data are presented as mean ± SEM. *P* values: ^*^p < 0.05, ^**^p < 0.01, ^***^p < 0.001.

Based on these findings, we hypothesized that PFKFB3 acted as an adaptor protein in promoting the interaction of JunB its E3 ubiquitin ligase Nedd4. To investigate the relationship between PFKFB3 and Nedd4, we performed Co‐IP assays and observed that PFKFB3 could interact with Nedd4 both at the exogenous and endogenous level (Figure 8A, B).

Immunofluorescence co‐localization analysis also demonstrated clear co‐localization between PFKFB3 and Nedd4 (Figure 8C, D). Notably, we observed that knock‐down of PFKFB3 weakened the interaction of JunB and Nedd4, while exogenously expressed PFKFB3 enhanced the interaction of JunB and Nedd4 (Figure 8E), indicating that PFKFB3 promoted the interaction of JunB and Nedd4.

In addition, we found that PFKFB3 regulates the expression of Nedd4, as overexpression of PFKFB3 increased Nedd4 levels, and their expression levels showed a positive correlation (Figure S7A–D). In addition, silence of PFKFB3 or Nedd4 significantly attenuated the ubiquitination of JunB (Figure 8F). Together, these results demonstrated that PFKFB3 acted as a scaffold protein in bridging JunB and Nedd4, which was critical to JunB degradation.

We supplemented the expression of PFKFB3 and JunB during human muscle atrophy, and clarified the negative correlation between them in the human body. Furthermore, we extracted the atrophied muscles of patients after anterior cruciate ligament injury surgery, extracted the muscle proteins, conducted IP verification, and further clarified the relationships between PFKFB3, Nedd4 and JunB in human tissue samples. This suggests that this molecular mechanism is also applicable in the human body (Figure S3D, E).

### 2.9 JunB Suppresses PFKFB3 Expression by Binding to Its Promoter

Interestingly, we also found that JunB regulates PFKFB3. The qRT‒PCR result revealed that JunB overexpression led to a reduction in PFKFB3 expression, while JunB knockdown led to an increase in PFKFB3 expression (Figure 8G). These results indicate that JunB negatively regulates PFKFB3.

We determined that JunB may regulate PFKFB3 expression by binding to its promoter. Thus, we searched the musculus ChIP‐seq REMAP database and found that JunB binds to the PFKFB3 promoter, suggesting that JunB regulates PFKFB3 expression directly. Furthermore, we predicted regulation patterns by using the JASPAR database and found that JunB, as a transcription factor that binds to specific AP‐1 site (5'‐TGAGTCA‐3'), binds mainly to region 1 (1252 to 1264 bp with respect to the transcription start site (TSS)) and region 2 (617 to 629 bp with respect to the TSS) (Figure 8H).

Homology analysis showed that, compared with region 1, region 2 was highly conserved across species. Luciferase reporter assays demonstrated that JunB regulates PFKFB3 primarily by targeting region 2, and overexpression of JunB significantly increased this transcriptional activity (Figure 8I). Notably, binding and regulatory activity were also observed at region 1, albeit to a lesser extent than at region 2. Consistent with these findings, endogenous ChIP assays confirmed that JunB predominantly occupies region 2, while showing detectable binding to region 1 (Figure 8J, K). Together, these results suggest that JunB regulates PFKFB3 expression most likely through coordinating interactions with both region 1 and region 2 of the PFKFB3 promoter. Therefore, we identified a positive feedback loop formed by PFKFB3–Nedd4–JunB.

### 2.10 Targeting the PFKFB3–Nedd4–JunB Axis Inhibits Muscle Atrophy

**FIGURE 9:**
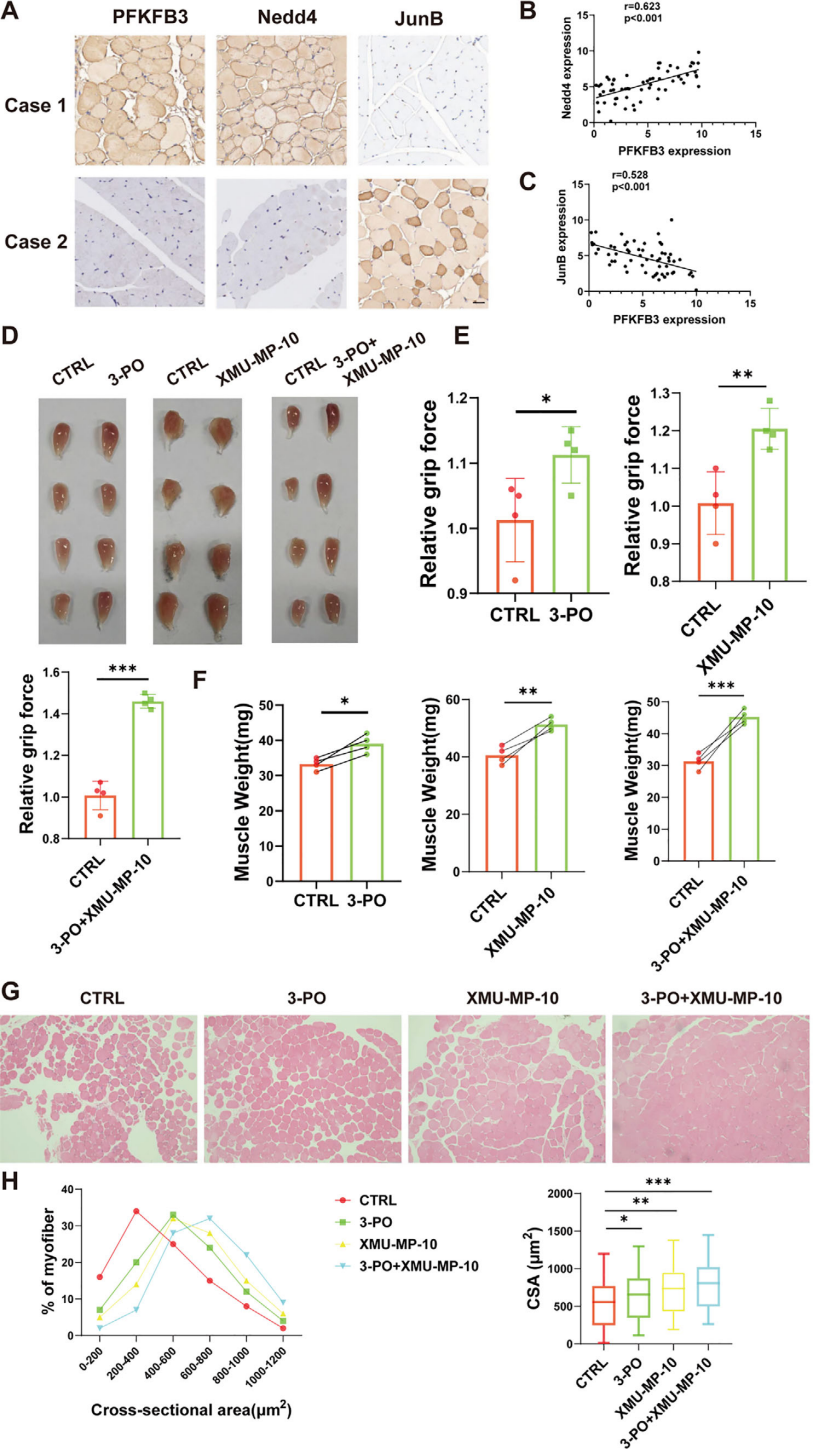
Targeting the PFKFB3–Nedd4–JunB axis alleviates muscle atrophy. (A) Immunohistochemical staining of PFKFB3, Nedd4, and JunB in muscle tissues (scale bar, 100 µm). (B, C) Correlation analyses between PFKFB3 and Nedd4 (B), and PFKFB3 and JunB (C). (D) Representative TA muscle images following treatment with 3‐PO, XMU‐MP‐10, or combination. (E, F) Grip strength (E) and TA muscle mass (F). (G) H&E staining of muscle sections. (H) Quantification of CSA. All data are presented as mean ± SEM. *P* values: ^*^p < 0.05, ^**^p < 0.01, ^***^p < 0.001.

To verify the in vivo role of the PFKFB3–Nedd4–JunB axis, we established a disuse‐induced muscle atrophy mouse model. Immunohistochemical analysis showed that the expression levels of PFKFB3 and Nedd4 were positively correlated with muscle atrophy, whereas JunB expression was negatively correlated (Figure 9A–C).

We then treated mice with 3‐PO (a PFKFB3 enzymatic activity inhibitor) and XMU‐MP‐10 (a specific Nedd4 small‐molecule inhibitor). By assessing grip strength, muscle mass, and muscle fiber cross‐sectional area, we found that treatment with XMU‐MP‐10 alone showed a more pronounced improvement in muscle atrophy compared with treatment with 3‐PO alone. Moreover, the combined treatment with 3‐PO and XMU‐MP‐10 showed the most significant therapeutic effect (Figure 9D–H). We conducted additional experiments in the gastrocnemius muscle to verify the role of the PFKFB3 axis in slow muscles (Figure S9A, B).

These results indicate that targeting the PFKFB3–Nedd4–JunB axis can significantly inhibit muscle atrophy in vivo. Simultaneous targeting of both PFKFB3 and Nedd4 showed the best effect. These preliminary in vivo findings suggest that co‐inhibition of PFKFB3 and Nedd4 by 3‐PO and XMU‐MP‐10 could serve as a potential starting point for developing therapeutic interventions against disuse‐induced muscle atrophy, though further pharmacological and translational validation is required.

## 3 Discussion

Muscle atrophy significantly impairs motor function, reduces quality of life, and increases fall risk. Although some progress has been made, effective treatment options are still lacking. PFKFB3 is a muscle‐enriched protein linked to muscle atrophy [19, 24]. Jin L et al. reported that PFKFB3 attenuates ventricular hypertrophy by promoting K6‐linked ubiquitination and stability of OPA1 [25]. In contrast, Wen T et al. found that PFKFB3 induces myocardial dysfunction via inflammation and apoptosis [12]. In this study, we demonstrate that PFKFB3 is highly expressed in disuse‐induced muscle atrophy and serves as a key driver of the condition. Specifically, we show that PFKFB3 promotes muscle atrophy by mediating ubiquitin‐dependent degradation of JunB.

Altered metabolism is also closely associated with muscle atrophy [5, 26]. A shift toward glycolytic metabolism occurs during atrophy, and enhanced glycolysis worsens the condition. Clara B et al. showed that ISG15 inhibits cardiac atrophy by suppressing glycolysis [8]. Tomoyasu S et al. found that glycolysis inhibition ameliorates denervation‐induced muscle atrophy in mice [27]. Together, these studies underscore a strong link between glycolysis and muscle atrophy, pointing to glycolysis inhibition as a promising therapeutic approach. Consistent with this, we confirm that inhibiting the enzymatic activity of PFKFB3 alleviates muscle atrophy. Notably, suppressing PFKFB3 expression produced a more potent anti‐atrophy effect. This suggests that PFKFB3 drives muscle atrophy through both glycolytic and non‐glycolytic mechanisms.

PFKFB3 has been shown to localize to the nucleus, indicating non‐canonical functions beyond glycolysis. For example, Lu W et al. discovered that nuclear PFKFB3 inhibits CDK4, reducing cisplatin‐induced renal tubule cell apoptosis and kidney injury in mice [11]. Wen‐K et al. reported that PFKFB3 is mainly nuclear in hepatocellular carcinoma cells, where it promotes tumor growth and DNA repair [10]. Similarly, we observed predominant nuclear localization of PFKFB3 in muscle cells, supporting a non‐metabolic role. We further demonstrated that PFKFB3 interacts with the transcription factor JunB in the nucleus, reducing JunB protein stability and accelerating muscle atrophy. This finding broadens the functional scope of PFKFB3 in cellular regulation.

In this study, we demonstrated that PFKFB3 acts as a nuclear scaffold to facilitate the interaction between Nedd4 and JunB, thereby promoting JunB ubiquitination. However, we also observed that PFKFB3 overexpression independently increases overall Nedd4 protein levels. This reveals a previously unappreciated mechanistic complexity, indicating that PFKFB3 accelerates JunB degradation through at least two co‐existing mechanisms: (i) the scaffold‐mediated co‐recruitment of Nedd4 to JunB, and (ii) the up‐regulation of Nedd4 protein abundance. In the current study, we did not quantitatively uncouple these two effects to determine their relative contributions to JunB degradation. Future experiments utilizing targeted PFKFB3 scaffold‐mutants—which specifically lack the Nedd4‐binding interface but maintain normal expression and structural integrity—will be essential. By comparing these mutants with wild‐type PFKFB3 under conditions where Nedd4 abundance is tightly controlled, we could explicitly distinguish and quantify the independent contributions of the scaffolding function vs. the protein‐upregulation effect on JunB stability.

Nedd4, a HECT‐type E3 ubiquitin ligase, is known to contribute to muscle atrophy. Its classic role involves ubiquitinating target proteins to control their stability [28, 29]. Robert D et al. identified Nedd4 as a critical ubiquitin ligase in disuse atrophy, promoting ubiquitination and degradation of PDLIM7 [30]. Our study also establishes Nedd4 as a key player in muscle atrophy. Surprisingly, we found that Nedd4 requires a scaffold protein for optimal activity. PFKFB3 serves as this scaffold, enhancing Nedd4‐mediated ubiquitination. This mechanism represents a novel mode of action for Nedd4 [23, 31, 32, 33], and the structural basis of this interaction merits further study. These results indicate that Nedd4 influences atrophy not only through direct substrate targeting but also via scaffold‐assisted regulation.

While our in vivo pharmacological results demonstrate that dual inhibition of PFKFB3 and Nedd4 significantly alleviates disuse‐induced muscle atrophy, the systemic administration or potential systemic leakage of these inhibitors warrants careful consideration. In particular, Nedd4 is an essential ubiquitin ligase with well‐characterized physiological roles far beyond skeletal muscle homeostasis. Notably, Nedd4 regulates surface expression of the epithelial sodium channel (ENaC) in renal and pulmonary epithelia, thereby playing a critical role in sodium balance, fluid homeostasis, and blood pressure regulation [34, 35]. Furthermore, Nedd4‐mediated ubiquitination is actively involved in maintaining cardiac physiology, where its disruption has been linked to cardiac dysfunction and maladaptive ventricular hypertrophy. Consequently, although our study utilized localized intramuscular injection of XMU‐MP‐10 to maximize regional bioavailability and limit systemic exposure, the potential off‐target systemic risks—including blood pressure perturbations, renal salt‐wasting, or subclinical cardiac toxicity—cannot be completely ruled out. Future translational efforts must prioritize comprehensive pharmacokinetic and systemic toxicity evaluations, or develop muscle‐targeted delivery systems (such as myotropic antibody‐drug conjugates or muscle‐specific nanoparticle carriers) to safely translate this combination strategy into clinical therapy.

Notably, JunB mRNA levels remain largely unchanged during muscle atrophy, as previously reported [36]. Our data are consistent with this observation. However, we detected a significant decrease in JunB protein levels, indicating that JunB is regulated post‐translationally in atrophy.

JunB is known to suppress atrophy by interfering with FoxO3 binding to the promoters of Atrogin‐1 and MuRF1, thereby repressing proteasomal degradation [13]. JunB has also been linked to glycolytic regulation. Interestingly, we found that JunB suppresses glycolysis and inhibits PFKFB3. This implies that JunB may promote muscle hypertrophy by modulating glycolytic flux. This mechanism is distinct from the canonical Akt/mTOR signaling pathways [37]. These findings highlight the therapeutic potential of targeting PFKFB3 to stabilize JunB in muscle atrophy.

We show that dual inhibition of PFKFB3 enzyme activity and Nedd4 function effectively counteracts disuse‐induced atrophy. We speculate that the inflammatory milieu in sarcopenia may attenuate the contribution of PFKFB3‐driven glycolysis [38]. This suggests that inflammatory signals may modulate PFKFB3 activity, a possibility that requires further study.

We show that dual inhibition of PFKFB3 enzyme activity and Nedd4 function effectively counteracts disuse‐induced atrophy. Moreover, PFKFB3 targeting also shows efficacy in other atrophy contexts, such as sarcopenia. However, the benefit was less pronounced in sarcopenia than in disuse atrophy. We speculate that the inflammatory milieu in sarcopenia may attenuate the contribution of PFKFB3‐driven glycolysis. This suggests that inflammatory signals may modulate PFKFB3 activity, a possibility that requires further study.

In summary, we have identified a key regulatory loop in disuse‐induced muscle atrophy: PFKFB3 promotes Nedd4‐mediated ubiquitination and degradation of JunB, while JunB transcriptionally suppresses PFKFB3 expression. This PFKFB3–JunB–Nedd4 feedback loop plays a central role in pathology and represents a highly promising target for treating disuse‐induced muscle atrophy.

## 4 Conclusion

This study uncovers a noncanonical, nuclear function of PFKFB3 in skeletal muscle atrophy. PFKFB3 promotes JunB degradation by scaffolding its interaction with Nedd4, thereby contributing to atrophic progression. The reciprocal regulation between PFKFB3 and JunB establishes a feedback loop that may reinforce muscle wasting. Importantly, disruption of this axis mitigates muscle atrophy in vivo, highlighting its potential as a therapeutic target.

## 5 Experimental Section

### 5.1 Cell Culture and Differentiation

C2C12 myoblasts and HEK293T cells were obtained from the American Type Culture Collection (ATCC) and cultured according to standard protocols. C2C12 cells were maintained in Dulbecco's Modified Eagle Medium (DMEM) supplemented with 10% fetal bovine serum (FBS) and 1% penicillin–streptomycin. To induce myogenic differentiation, cells were switched to DMEM containing 2% horse serum upon reaching confluence, and the medium was replaced every 2 days. All cells were cultured at 37°C in a humidified incubator with 5% CO_2_.

### 5.2 Plasmid Construction and Generation of Stable Cell Lines

The plasmids pLVX‐PFKFB3‐FLAG, pLKO.1‐shPFKFB3, pLVX‐JunB‐HA, pLKO.1‐shJunB, pLVX‐Nedd4‐Myc, and pLKO.1‐shNedd4 were synthesized commercially. The shRNA target sequences were as follows:

- shPFKFB3#1: 5′‐GCACCG AAGATGATACCGTTT‐3′
- shPFKFB3#2: 5′‐GCTGCCTAAATATTGAACTTA‐3′
- shJunB#1: 5′‐GAGTCGCCTTTCTACTACGAC‐3′
- shJunB#2: 5′‐GCATCACCTCCGACCTTTA‐3′
- shNedd4#1: 5′‐GCAGCAACAACATTAGTTAGA‐3′
- shNedd4#2: 5′‐CGTGGTGAATTGCTATACAAA‐3′

Lentiviral particles were produced by co‐transfecting HEK293T cells with transfer plasmids, psPAX2, and pMD2.G using Lipofectamine 3000. Viral supernatants were collected at 24, 48, and 72 h post‐transfection, filtered, and used to infect target cells in the presence of Polybrene. Stable knockdown or overexpression cell lines were established via antibiotic selection using puromycin or hygromycin.

### 5.3 RNA Extraction and Quantitative Real‐Time PCR (qRT‐PCR)

Total RNA was extracted using a commercial RNA isolation kit. RNA concentration was measured using a spectrophotometer, and cDNA was synthesized using reverse transcription reagents. Quantitative PCR was performed using SYBR Green Master Mix. Vinculin was used as an internal control, and relative expression levels were calculated using the 2^−ΔΔCt method. Primer sequences are listed in Table S1.

### 5.4 Western Blot Analysis

Total protein was extracted using RIPA lysis buffer supplemented with PMSF and phosphatase inhibitors. Protein concentrations were determined using a BCA assay. Equal amounts of protein were subjected to SDS‐PAGE and transferred onto PVDF membranes. Membranes were blocked with 5% non‐fat milk and incubated with primary antibodies overnight at 4°C, followed by HRP‐conjugated secondary antibodies. Signals were detected using enhanced chemiluminescence (ECL) and quantified using ImageJ software. We chose Vinculin as the internal control, and we also verified the expression stability of Vinculin under all experimental conditions (Protein quantification was performed prior to detection) (Figure S5F). The antibodies used in the research are summarized in Table S2.

### 5.5 Animal Experiments

All animal procedures were approved by the Institutional Animal Care and Use Committee. Six‐week‐old male C57BL/6 mice were maintained under specific pathogen‐free (SPF) conditions.

A unilateral hindlimb immobilization model was established to induce disuse muscle atrophy. The right hindlimb was immobilized in an extended position for 1, 2, or 4 weeks, while the contralateral limb served as the internal control. Grip strength was measured prior to tissue collection. Tibialis anterior (TA), gastrocnemius (GAST), and quadriceps (QUAD) muscles were harvested, weighed, and processed for further analysis.

Muscle‐specific overexpression of PFKFB3 was achieved by intramuscular injection of AAV9 carrying sgRNA targeting PFKFB3 into the TA muscle and GAST muscle.

Six‐week‐old male C57BL/6 mice subjected to unilateral hindlimb immobilization were randomly assigned to four experimental groups: vehicle control (CTRL), PFKFB3 inhibitor (3‐PO), specific Nedd4 inhibitor (XMU‐MP‐10), and combined treatment (3‐PO + XMU‐MP‐10).

All drug interventions were administered via local intramuscular (IM) injection directly into the tibialis anterior (TA) muscle to minimize systemic exposure and off‐target toxicity. Specifically, 3‐PO (MedChemExpress, HY‐13323) was dissolved in sterile PBS containing 5% DMSO, 40% PEG300, 5% Tween‐80, and 50% sterile saline, and was administered at a dose of 1.5 mg/kg body weight (in a total volume of 20 µL per injection site) three times per week (every 2–3 days) throughout the 4‐week immobilization period. XMU‐MP‐10 (MedChemExpress, HY‐128456) was formulated in the same aqueous vehicle system and intramuscularly injected at a dose of 2.0 mg/kg body weight twice per week over the 4‐week period. For the combination therapy group, both inhibitors were co‐administered at their respective dosages and schedules, while the CTRL group received an equivalent volume (20 µL) of the vehicle solution. Injection sites were carefully alternated across different aspects of the TA muscle belly using a 30‐gauge insulin syringe to avoid local tissue trauma. At the conclusion of the 4‐week intervention, hindlimb grip strength was evaluated, and TA muscles were harvested for subsequent morphological, biochemical, and histological analyses.

### 5.6 GST Pull‐Down Assay

GST‐tagged fusion proteins were expressed in E. coli and purified using glutathione‐Sepharose beads. Cell lysates containing potential interacting proteins were incubated with immobilized GST fusion proteins. After extensive washing, bound proteins were eluted, separated by SDS‐PAGE, and analyzed by Western blot.

### 5.7 Dual‐Luciferase Reporter Assay

The PFKFB3 promoter and its mutant constructs were cloned into the pGL3‐basic vector. C2C12 cells were co‐transfected with reporter plasmids and Renilla luciferase control plasmids. Luciferase activity was measured 48 h post‐transfection using a dual‐luciferase reporter assay system.

### 5.8 Chromatin Immunoprecipitation (ChIP)

ChIP assays were performed using a commercial kit according to the manufacturer's instructions. Chromatin was immunoprecipitated using anti‐JunB antibody, and purified DNA was analyzed by PCR.

### 5.9 Untargeted Metabolomics Analysis

Frozen muscle tissues were homogenized in methanol/acetonitrile/water (2:2:1) extraction buffer. Samples were analyzed by UHPLC coupled with high‐resolution mass spectrometry under both positive and negative ion modes. Data were processed for peak detection, alignment, and metabolite identification using public databases (HMDB, KEGG, mzCloud). Multivariate statistical analyses including PCA and OPLS‐DA were performed.

### 5.10 Co‐Immunoprecipitation (Co‐IP)

Cell lysates were incubated with specific antibodies followed by Protein A/G magnetic beads. Immunocomplexes were washed, eluted, and analyzed by Western blot.

### 5.11 Immunofluorescence (IF)

Cells were fixed with paraformaldehyde, permeabilized, and blocked with serum. After incubation with primary and fluorescent secondary antibodies, nuclei were stained with DAPI. Images were acquired using a confocal microscope.

### 5.12 Isolation of Muscle Satellite Cells

Skeletal muscles were dissected, minced, and digested with collagenase and Dispase II. The resulting cell suspension was filtered, centrifuged, and subjected to pre‐plating to remove fibroblasts. Satellite cells were cultured in growth medium supplemented with FBS and growth factors.

### 5.13 Nuclear and Cytoplasmic Fractionation

Cells were lysed in cytoplasmic extraction buffer, and nuclei were isolated by centrifugation. Nuclear proteins were subsequently extracted using high‐salt buffer. GAPDH and Histone H3 were used as cytoplasmic and nuclear markers, respectively.

### 5.14 Histology and Immunohistochemistry

Paraffin‐embedded tissue sections were subjected to hematoxylin and eosin (H&E) staining for morphological analysis. For immunohistochemistry, antigen retrieval was performed followed by incubation with primary antibodies and HRP‐conjugated secondary antibodies. Signals were visualized using DAB substrate.

### 5.15 In Vitro Ubiquitination Assay

An in vitro ubiquitination assay was performed using purified recombinant proteins to determine whether Nedd4 directly ubiquitinates JunB. Recombinant human E1 ubiquitin‐activating enzyme (UBE1), E2 ubiquitin‐conjugating enzyme (UbcH5c), ubiquitin (Ub), recombinant Nedd4 protein, and recombinant JunB protein were incubated in ubiquitination reaction buffer containing 50 mm Tris‐HCl (pH 7.5), 5 mm MgCl_2_, 2 mm ATP, and 1 mm DTT in a final volume of 30 µL. Unless otherwise indicated, each reaction contained 100 ng E1, 250 ng UbcH5c, 500 ng recombinant Nedd4, 500 ng recombinant JunB, and 5 µg ubiquitin.

The reaction mixtures were incubated at 30°C for 60 min with gentle agitation. Reactions were terminated by the addition of 4× SDS loading buffer followed by boiling at 95°C for 5 min. Proteins were separated by SDS‐PAGE and transferred onto PVDF membranes for immunoblotting.

### 5.16 Oil Red O Staining

Neutral lipid accumulation in skeletal muscle was evaluated by Oil Red O (ORO) staining. Fresh skeletal muscle tissues were embedded in optimal cutting temperature (OCT) compound and rapidly frozen in liquid nitrogen‐cooled isopentane. Serial cryosections (8–10 µm thick) were prepared using a cryostat and mounted onto glass slides. Sections were air‐dried for 15 min at room temperature and fixed with 4% paraformaldehyde for 10 min. After washing three times with phosphate‐buffered saline (PBS), sections were equilibrated in 60% isopropanol for 1 min.

Oil Red O working solution was freshly prepared by diluting a 0.5% Oil Red O stock solution in isopropanol with distilled water at a ratio of 3:2 (v/v), followed by filtration through a 0.22‐µm membrane. Tissue sections were incubated with the working solution for 15 min at room temperature. Excess dye was removed by briefly rinsing the sections in 60% isopropanol, followed by extensive washing with distilled water. Nuclei were counterstained with hematoxylin for 1 min, rinsed under running tap water, and mounted with an aqueous mounting medium.

### 5.17 Human Samples

Muscle tissue samples from human bodies were taken from patients who underwent surgery for anterior cruciate ligament injury at the Department of Sports Medicine of the Eighth Affiliated Hospital of Sun Yat‐sen University. No written consent has been obtained from the patients as there is no patient identifiable data included in this research.

### 5.18 Statistical Analysis

All data are presented as mean ± SEM from at least three independent experiments. Statistical analyses were performed using GraphPad Prism 8. Differences between two groups were assessed using unpaired two‐tailed Student's t‐test, while multiple group comparisons were performed using one‐way or two‐way ANOVA. Correlations were analyzed using Pearson's r (correlation coefficient). A *p* value < 0.05 was considered statistically significant.

## Author Contributions

Project conception and experiment design. L.H. and M.M. Experimental operation. L.Y., W.W., Y.B., Z.J., Y.H., M.R., R.Y., and Y.W. Sample collection. Y.H., Y.C., and G.Y. Data analysis. L.Y., M.M., and W.W. Writing – original draft. L.Y and W.W. Writing – review and editing. L.Y. and M.M. All experiments were conducted under the supervision of L.H. All authors contributed to design development, data interpretation, and manuscript editing work. Some experimental schemes were created with BioRender.com ( https://biorender.com/ ), and all were con‐firmed of publication and licensing rights.

## Funding

This study was supported by grants from National Natural Science Foundation of China (82203740), Natural Science Foundation of Guangdong Province (2023A1515010552), Shenzhen Science and Technology Program (JCYJ20230807111307015), Futian Healthcare Research Project (FTWS2025023) and Outstanding Medical lnnovation Talent Program of the Eighth Affiliated Hospital of Sun Yat‐sen University (YYQ202103).

## Conflicts of Interest

The authors declare no conflicts of interest.

## Supporting information




**Supporting File 1**: advs77854‐sup‐0001‐SuppMat.docx.

**Supporting File 2**: advs77854‐sup‐0002‐TableS1.docx.

**Supporting File 3**: advs77854‐sup‐0003‐TableS3.docx.

## Data Availability

The data underlying this article will be shared on reasonable request to the corresponding author. The RNA‐seq data could be obtained from the Gene Expression Omnibus (GEO) DataBase(https://www.ncbi.nlm.nih.gov) with an accession number GSE211204. The processed data and analysis codes are available upon request from the corresponding author.
