# Supplementary material for: Nuclear Translocation of PFKFB3 Promotes Disuse‐Induced Muscle Atrophy via Scaffolding Nedd4‐Mediated JunB Ubiquitination
Source: Adv Sci (Weinh). 2026 Sep 16:e77854. Online ahead of print. doi: 10.1002/advs.77854 (PMC13583119; doi:10.1002/advs.77854)
Supplement: Supplementary file 1 [file ADVS-9999-e77854-s001.docx]

**Figure S1. Successful establishment of the disuse-induced muscle atrophy model.**
(A) Expression levels of PFKFB3 across groups in the GSE211204 dataset.
(B) Schematic of unilateral hindlimb immobilization in mice.
(C–D) Representative images of gastrocnemius muscle (GAST) (C) and quantification of muscle weight (D).
(E–F) Hematoxylin and eosin (H&E) staining of GAST sections (E) and quantification of myofiber cross-sectional area (CSA) (F).
(G–H) Representative images of quadriceps muscle (QUAD) (G) and quantification of muscle weight (H).
(I–J) H&E staining of QUAD sections (I) and CSA quantification (J).
(K) mRNA levels of PFKFB3 in muscle satellite cells and non-satellite cells.
All data are presented as mean ± SEM. P values: *P < 0.05, **P < 0.01**,** ***P **< 0.001**.

**Figure S2. Inhibiting the enzymatic activity of PFKFB3 can partially alleviate muscle atrophy.
(A) The results of immunofluorescence staining of MyHC in the C2C12 myotube of the control group, the group overexpressing PFKFB3, and the group overexpressing PFKFB3 with inhibitor 3-PO added simultaneously are presented. (B) Quantitative analysis of the diameter of muscle tubes using immunofluorescence staining in (A). (C–E) mRNA levels (C) and protein levels (D–E) of MyHC, MuRF1, and Atrogin-1 in C2C12 myotubes treated with 3-PO compared with PFKFB3 overexpression alone.**

All data are presented as mean ± SEM. P values: *P < 0.05, **P < 0.01**,** ***P **< 0.001**.

**Figure S3. PFKFB3 localizes to the nucleus and induces muscle atrophy in human samples.
(A) Immunofluorescence staining of PFKFB3 in human muscle cells.
(B) mRNA levels of MyHC, MuRF1, and Atrogin-1 measured by qPCR.
(C) Protein levels of MyHC, MuRF1, and Atrogin-1 detected by Western blotting.**

(D) **The mRNA expression levels of PFKFB3 and JunB in human muscle atrophy samples (n=6).**
(E) **An IP experiment was conducted using the antibody against PFKFB3 on the samples of human muscle atrophy, to verify the binding with JunB and Nedd4.**

**All data are presented as mean ± SEM. P values: *P < 0.05, **P < 0.01, ***P < 0.001.**

**Figure S4. JunB promotes muscle hypertrophy.
(A) Coomassie Brilliant Blue staining.
(B–C) Immunofluorescence staining of MyHC following JunB knockdown (B) and quantification of myotube length and fusion index compared with control (C).
(D–E) mRNA levels of MyHC, MuRF1, and Atrogin-1 in JunB-overexpressing (D) and JunB-knockdown (E) groups.
(F–G) Protein levels of MyHC, MuRF1, and Atrogin-1 in JunB-overexpressing cells.
(H–I) Protein levels of MyHC, MuRF1, and Atrogin-1 in JunB-knockdown cells.
All data are presented as mean ± SEM. P values: *P < 0.05, **P < 0.01, ***P < 0.001.**

**Figure S5. PFKFB3 regulates muscle atrophy through JunB.
(A) Effect of PFKFB3 overexpression on JunB protein levels.
(B) Effect of PFKFB3 knockdown on JunB protein levels.
(C) Western blot analysis of shPFKFB3 combined with shJunB.
(D) Quantification of protein levels shown in (C).**

**(E) The expression levels of PFKFB3 and JunB in mice at fixed time points of 0, 7, 14, and 28 days.**

**(F) The muscle proteins of each group were quantitatively normalized, and then the protein expression level of Vinculin under various experimental conditions was detected.**

**All data are presented as mean ± SEM. P values: *P < 0.05, **P < 0.01, ***P < 0.001.**

**Figure S6. Nedd4 does not affect JunB mRNA levels.
(A–B) Changes in JunB mRNA levels upon Nedd4 knockdown(A) and overexpression (B).**

(C) **Knockdown of Nedd4 reduces the ubiquitination level of JunB.**

**All data are presented as mean ± SEM. ns, not significance.**

**Figure S7. PFKFB3 regulates Nedd4 protein levels.
(A–B) Effects of PFKFB3 overexpression on Nedd4 protein levels.
(C–D) Effects of PFKFB3 knockdown on Nedd4 protein levels.**

**All data are presented as mean ± SEM. P values: *P < 0.05, **P < 0.01, ***P < 0.001.**

**Figure S8. The effects of overexpression of PFKFB3 on fast muscle fibers and slow muscle fibers.**

（A） **Immunofluorescence staining of MyHC-I type muscle fibers (slow muscle fibers) in the gastrocnemius muscle with overexpressed PFKFB3, as well as quantitative analysis of the cross-sectional area of the muscle fibers.**
（B） **Immunofluorescence staining of MyHC-II type muscle fibers (fast muscle fibers) in the anterior tibial muscle with overexpressed PFKFB3, as well as quantitative analysis of the cross-sectional area of the muscle fibers.**

**All data are presented as mean ± SEM. P values: *P < 0.05, **P < 0.01, ***P < 0.001.**

**Figure S9. The therapeutic effect of targeting the PFKFB3 axis in the gastrocnemius muscle.**

(A) **The effect of overexpressing PFKFB3 in the gastrocnemius muscle on muscle mass.**
(B) **The atrophied gastrocnemius muscles were treated with 3-PO, XMU-MP-10 and 3-PO + XMU-MP-10 respectively, and the effects on the muscle mass were examined.**

**All data are presented as mean ± SEM. P values: *P < 0.05, **P < 0.01, ***P < 0.001.**

**Figure S10. Overexpression of PFKFB3 by AAV in the C2C12 myotube leads to myotube atrophy.**

(A) **By overexpressing PFKFB3 using AAV in the C2C12 myotubes (AAV-oePFKFB3), compared with the virus-free group (CTRL) and the AAV control group (AAV-Mock), the results of MyHC immunofluorescence staining of the myotubes and the statistics of myotube diameters were obtained.**

**Figure S11. Oil Red O staining of frozen sections of muscle tissue.**

(A) **Frozen section of muscle tissue with overexpressed PFKFB3 and the oil red O staining results of the control group.**

 **Figure S12. Original blot figure.**

 **Figure S13. Original blot figure.**

 **Figure S14. Original blot figure.**

 **Figure S15. Original blot figure.**

 **Figure S16. Original blot figure.**

 **Figure S17. Original blot figure.**
