# Supplementary material for: Nuclear Translocation of PFKFB3 Promotes Disuse‐Induced Muscle Atrophy via Scaffolding Nedd4‐Mediated JunB Ubiquitination
Source: Adv Sci (Weinh). 2026 Sep 16:e77854. Online ahead of print. doi: 10.1002/advs.77854 (PMC13583119; doi:10.1002/advs.77854)
Supplement: Supplementary file 2 [file ADVS-9999-e77854-s002.docx]

| Supplementary Table 1  Primer list designed for qRT-PCR | | |
| --- | --- | --- |
| Gene | Forward Primer(5’-3’) | Reverse Primer(3’-5’) |
| PFKFB3 | ATGCCCTGAGCAAGTTCGTA | AGTTCCTCACACACACCAGCA |
| JunB | CCGGATGTGCACGAAAATGG | TGTCTGATCCCTGACCCGAA |
| Nedd4 | TTCTGACAACGCCTCCAACA | TTCTTGGGTTTTCAGTCGGT |
| Atrogin-1 | CGGGGTTTGTTTTCAGCAGG | ACACAGACATTGCCTCCCAG |
| MuRF1 | GAGGGGCTACCTTCCTCTCA | TCCCAAAGTCAATGGCCCTC |
| Vinculin | TGGTCTAGCAAGGGCAATGA | CTCGTCACCTCATCAGAGGC |
| MyHC | CTTACTTGCTACCCTCAGGTGG | GGTCAGCCTTTCTGGTCACT |

Supplementary Table 1：The gene primers used in this study and their sequences.
