# Supplementary material for: Nuclear Translocation of PFKFB3 Promotes Disuse‐Induced Muscle Atrophy via Scaffolding Nedd4‐Mediated JunB Ubiquitination
Source: Adv Sci (Weinh). 2026 Sep 16:e77854. Online ahead of print. doi: 10.1002/advs.77854 (PMC13583119; doi:10.1002/advs.77854)
Supplement: Supplementary file 3 [file ADVS-9999-e77854-s003.docx]

| Supplementary Table 2 | |
| --- | --- |
| Antibody | source |

| PFKFB3 Antibody | Solarbio |
| --- | --- |
| JunB Antibody | Cell Signaling Technology |
| Nedd4 Antibody | Cell Signaling Technology |
| Vinculin Antibody | Signalway Antibody |
| MuRF1 Antibody | Cell Signaling Technology |
| Atrogin-1 Antibody | Cell Signaling Technology |
| MyHC Antibody | Cell Signaling Technology |
| GAPDH Antibody | Cell Signaling Technology |
| GST-tag Antibody | Cell Signaling Technology |
| HA-tag Antibody | Cell Signaling Technology |
| FLAG-tag Antibody | Cell Signaling Technology |
| His-tag Antibody | Abcam |

Supplementary Table 2：The antibodies used in this research and their sources.
